# Congress on School Hygiene

**Published:** 1907-08-24

**Authors:** 


### CONGRESS ON SCHOOL HYGIENE.

The second International Congress on School Hygiene, held at the University of London early in August, drew together about two thousand hygienists and educationists from almost every country of Europe. Among the subjects dealt with, the medical inspection of schools occupied a prominent place, a discussion on the subject being opened by Dr. Leslie Mackenzie, the medical inspector of the Local Government Board for Scotland. Dr. Mackenzie takes it for granted that the principle of medical inspection is accepted, and pointed out that it should be strictly practical, *i.e.* it should be limited to the health conditions which fit or unfit a child for school work, and should not include, except as an incident, details of merely scientific interest, such as anthropometrical and anthropological observations. He does not exclude, however, inquiry into the conditions of the home, if only for the information of the public health authority, which has a moderately complete control of the hygiene of the house. Dr. Méry, of Paris, who followed Dr. Mackenzie, includes the anthropometric examination among the necessary inquiries, in addition to the investigation of sight and hearing, and the general organic examination. Both gentlemen said that the assistance of the teacher is necessary for the satisfactory compilation of a health register, and Dr. Mackenzie said that he had found teachers thoroughly interested in the work.

A kindred subject is the co-ordination of doctor, nurse, and teacher in inspecting children at school. Here also the aid of the teacher is invoked, and Dr. Hayward, of Wimbledon, suggested that the teachers should keep a clinical thermometer in each class-room, and take the temperature of children whom they suspect of being ill, besides keeping a health record of their pupils. He also thinks that teachers should know the analysis of mental capacity, and the tests for this. Naturally he wishes that, to this end, teachers should be trained in hygiene. Dr. Forbes, of Cambridge, who dealt with the subject of the school nurse, wants to throw the greatest responsibility on her. He would allow pupils to be excluded from school on the nurse's diagnosis, and would even leave it to her to say when they should return after infectious illness. Mr. Sykes, of Bradford, a teacher of long experience, is rather sceptical about the advantages of the proposed medical inspection, basing his opinion on the facility with which the medical inspectors to the factories pass half-timers as fit for work—the average pass being about 98.6 per cent. of the candidates.

The papers read by several medical officers of health give proof, however, of a higher standard of professional rectitude than Mr. Sykes gives medical men credit for. Sir Shirley Murphy opened the proceedings of the section devoted to the consideration of infectious disease with a paper that showed the keenest appreciation of the need of medical supervision of even trivial cases of illness among school children. Both he and Dr. Niven, of Manchester, pointed out how often a great number of children might be seriously infected by a slight case, or even by a "carrier" who having no symptoms himself, had the power to infect susceptible persons. This would justify the exclusion of children from homes where there was infectious disease, and Dr. Niven pointed out that children suffering from diphtheritic rhinitis, post-scarlatinal or other, might be the cause of an outbreak of diphtheria without themselves suffering from the disease. Dr. F. J. Poynton, out-patient physician at the Children's Hospital in Great Ormond Street, spoke of the prevalence of rheumatism among children, with the consequent risk of heart-disease, and pleaded for the founding of convalescent homes where rheumatic children might get thorough rest, and, failing that, for careful consideration in the school of these children, who are delicate and need careful management. He also spoke of the mischief done by neglected teeth, chronic ear discharges, and adenoids.

Dr. Newsholme, medical officer of health for Brighton, spoke of the disadvantages of children under five years of age attending schools, a subject on which he holds very strong views. He contends that not only do the children gain nothing by being sent to school at three years of age or a little over, when they would develop better either at home or at a crêche, but these very young children were peculiarly liable to infectious diseases; both the attack rate and the death rate from measles, whooping-cough, diphtheria, and scarlet fever are higher among children under five. Dr. Niven attributes the lesser fatality among elder children in part to immunity gained by previous attacks; but Dr. Newsholme points out that there is a great advantage to children in even postponing the age at which they are attacked by any of these diseases, for not only are they less likely to be fatal, but the postponement diminishes the likelihood of subsequent attack. As he reckons the cost of the education of infants at £1,749,711, and the intellectual result is admittedly not great, there would seem to be sufficient reason for making the postponement of school attendance to the age of five, which is now optional, compulsory, and for providing crêches where necessary.

Dr. N. Bishop Harman, oculist to the London County Council blind schools, gave some interesting statistics about blind children. He estimated that 37 per cent. of the total blind are blinded by purulent ophthalmia of the newly born, a cause which is in its nature accidental, and which from the greater care that is now being taken in the training of midwives and nurses, may be expected to diminish. As these children are otherwise normal, they can be successfully taught and may be made self-supporting, whereas children who are blind through congenital disease are often mentally defective. The most difficult class are the partially blind, including the highly myopic. These children cannot be taught in ordinary schools, owing to the defects of vision, but Dr. Harman does not think that blind schools, where, according to the scheduled curriculum, they must read and write Braille, are the right places for them. "The children learn it with ease, for they are of intelligence; but no sooner is the teacher's back turned than they bow down their heads to *see* what they must perforce read by *feel*." He suggests that they should learn to read and write like normal children, save that scrolls of large type should be used as lesson-books, and writing be learned with blackboard and chalk, free-arm fashion. Dr. Bronner, of Bradford, recommended that the so-called "conscience clause" in the Vaccination Acts should be abolished, and those who publish misleading statements on vaccination be punished. He dreads a return to the high percentage of blindness due to small-pox which characterised our country before the days of Jenner. He also advises that the eyes of all school children should be periodically examined by an expert, a record kept of cases of defective vision, and glasses insisted on, at the parents' expense when practicable, but where these are too poor, to be given free.

Dr. Stackler, of Paris, spoke of the usefulness of examining chudren for aural as well as ocular defects, so that the children might be placed in class according to their hearing capacity—the most defective nearest the teacher, and at the right or left side according as one ear or the other was most severely affected. Dr. Marion Hunter emphasised that it was necessary that the teeth also should receive attention in schools; while Drs. H. Meredith Richards and Winifred Thorpe dealt with the municipal control of ringworm. Among the many other subjects dealt with, ventilation and the training of teachers in hygiene were prominent, and the interchange of views among the doctors and teachers present should not fail to be useful.

